# Unveiling the “Kebab” technique: A case report on a two-stage reconstruction method for repeated complex cranioplasty

**DOI:** 10.1097/MD.0000000000034963

**Published:** 2023-09-01

**Authors:** Liang-Jui Chiang, Jing-Wei Lee, Po-Hsuan Lee, Jung-Shun Lee

**Affiliations:** a Section of Plastic Surgery, Department of Surgery, National Cheng Kung University Hospital, College of Medicine, National Cheng Kung University, Tainan, Taiwan; b Section of Neurosurgery, Department of Surgery, National Cheng Kung University Hospital, College of Medicine, National Cheng Kung University, Tainan, Taiwan; c Department of Cell Biology and Anatomy, College of Medicine, National Cheng Kung University, Tainan, Taiwan.

**Keywords:** case report, cranioplasty, free flap reconstruction, infection, sinking skin flap syndrome

## Abstract

**Rationale:**

Cranioplasty after decompressive craniectomy provides brain protection and improves cerebral hemodynamics. However, recurrent infection and sinking skin flap syndrome after cranioplasty remain cumbersome complications that require a well-planned reconstruction strategy.

**Patient concerns:**

A 74-year-old man presented with traumatic subdural hematoma and underwent decompressive craniectomy. Cranioplasty using an original bone flap, bone cement with wires, and a titanium mesh were complicated and resulted in recalcitrant infection and sinking skin flap syndrome.

**Diagnoses:**

Recurrent infection and sinking skin flap syndrome post-cranioplasty.

**Interventions:**

We designed a two-stage “kebab” reconstruction technique using a combination of free latissimus dorsi myocutaneous flap and delayed non-vascularized free rib graft. A well-vascularized musculocutaneous flap can obliterate dead space in skull defects and reduce bacterial inoculation in deep infections. Subsequently, delayed rib grafts act as the scaffold to expand the sunken scalp flap.

**Outcomes:**

At the 3-year follow-up, the patient showed improvement in headache, without evidence of surgical site infection.

**Lessons:**

The novel “kebab” technique using a combination of a free myocutaneous flap and delayed rib graft can eliminate bacterial growth in infected calvarial defects, reverse sinking skin flap syndrome, and minimize potential donor-site morbidity, and is therefore suitable for patients who require multiple cranioplasties and are unable to withstand major reconstructions.

## 1. Introduction

Decompressive craniectomy is lifesaving after severe traumatic brain injury.^[1]^ Once brain swelling has subsided, cranioplasty is indicated to avoid ongoing neurological deficit.^[2,3]^ However, post-cranioplasty infection is a major complication, with an incidence ranging from 2% to 26%.^[4,5]^ Adequate debridement, bone graft removal, and prolonged antibiotic therapy are mandatory before secondary cranioplasty. Preventing recurrent infection after repeated cranioplasty remains challenging. Herein, we propose a novel two-stage free flap reconstruction, the “kebab” technique, to manage cranial defects with refractory infection and multiple cranioplasty.

## 2. Case report

### 2.1. Case description

**Figure 1.**
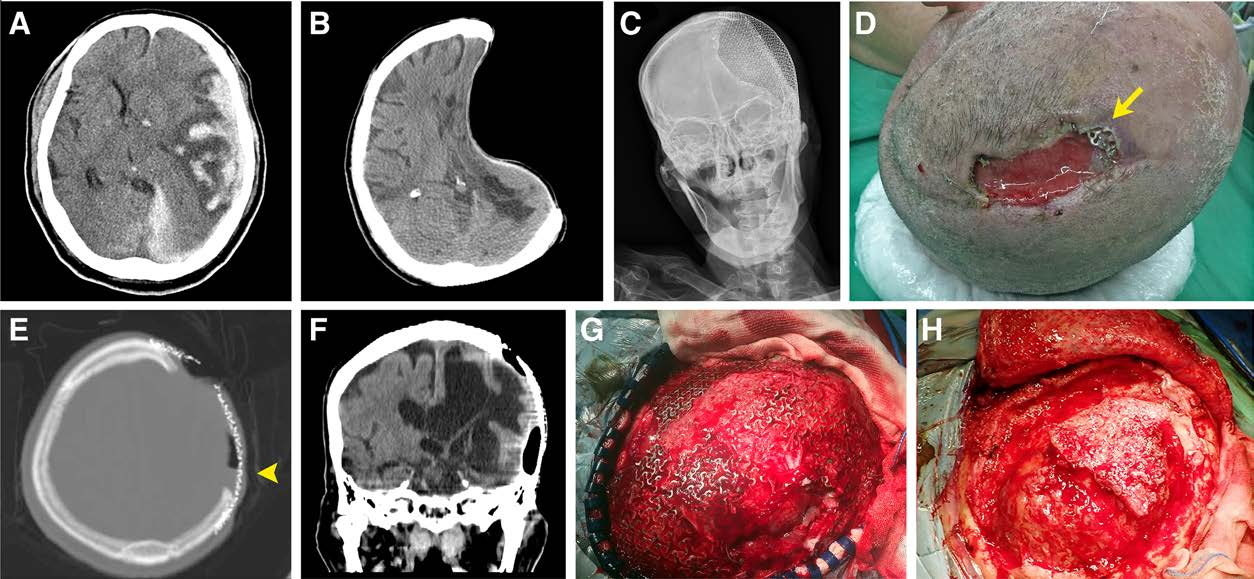
Cranioplasty with a sunken skin flap and refractory infection. (A) Brain computed tomographic (CT) scan showing left traumatic subdural hemorrhage with significant midline shift. (B) Severe paradoxical brain herniation after failed cranioplasty. (C) Restored calvarial contour after titanium mesh cranioplasty. (D) Poorly healed wound with mesh exposure (*yellow arrow*) 3 years later. (E) Axial and (F) coronal views from the CT scan revealing loculated fluid and air (*yellow arrowhead*) in the epidural space of the left frontotemporoparietal region, illustrated in different window settings. Pus accumulation and necrotic tissue were found (G) before and (H) after titanium mesh removal, resulting in a calvarial defect (15 × 15 cm) with dural exposure.

Written permission was obtained from the patient for the publication of the photos presented in this report. A 74-year-old man underwent decompressive craniectomy for traumatic subdural hemorrhage 15 years before (Fig. 1A) and cranioplasty with an autologous bone graft 2 months later. The patient’s neurological status was E4VaM5 with right hemiplegia. However, left temporal wound discharge with oxacillin-resistant *Staphylococcus aureus* infection was observed 1 month later.

Despite adequate debridement and close follow-up, epidural empyema still occurred after secondary cranioplasty with wire mesh and bone cement. During another cycle of intravenous antibiotic treatment and hyperbaric oxygen therapy, free latissimus dorsi (LD) and serratus anterior myo-osseous flap transfer was suggested, but his family refused the reconstruction because of its high invasiveness. The patient lived with a sunken scalp and chronic headache (Fig. 1B). Five years later, cranioplasty using a titanium mesh (Fig. 1C) was complicated with infection and mesh exposure again (Fig. 1D). Computed tomography of the brain revealed loculated fluid and air in the frontotemporoparietal epidural space, suggestive of an epidural abscess (Fig. 1E and F).

### 2.2. Surgical technique

**Figure 2.**
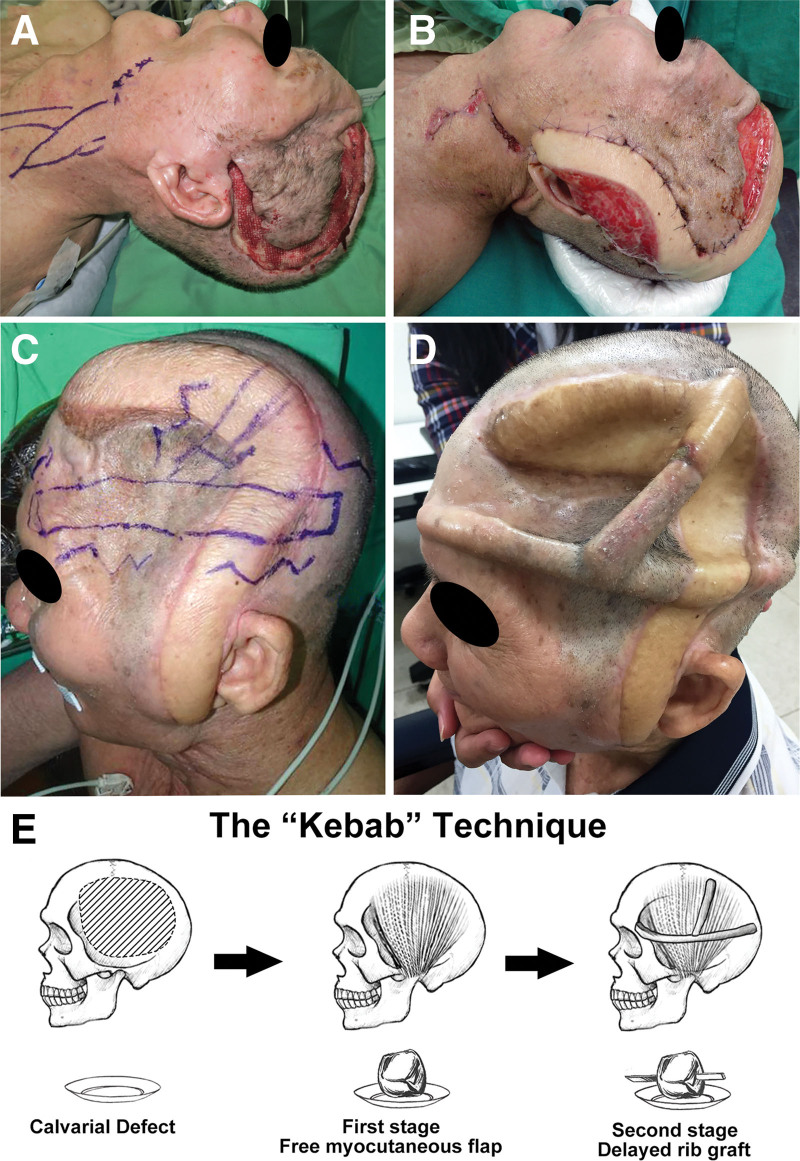
Preoperative photograph after implant removal and debridement, showing a crescent-shaped scalp defect over the depressed dura. (A) Planned recipient vessels were marked after Doppler assessment. (B) First stage of the “kebab” method: inset of free latissimus dorsi myocutaneous flap. The skin defects relative to the transferred muscle bulk were covered with skin graft. (C) The second stage of the “kebab” method with planned W-shaped incisions and perpendicular alignment of rib grafts. (D) Postoperative photograph 1 year after the “kebab” reconstruction, showing no active infection signs or flap necrosis. (E) Schematic illustration of the “kebab” technique.

**Figure 3.**
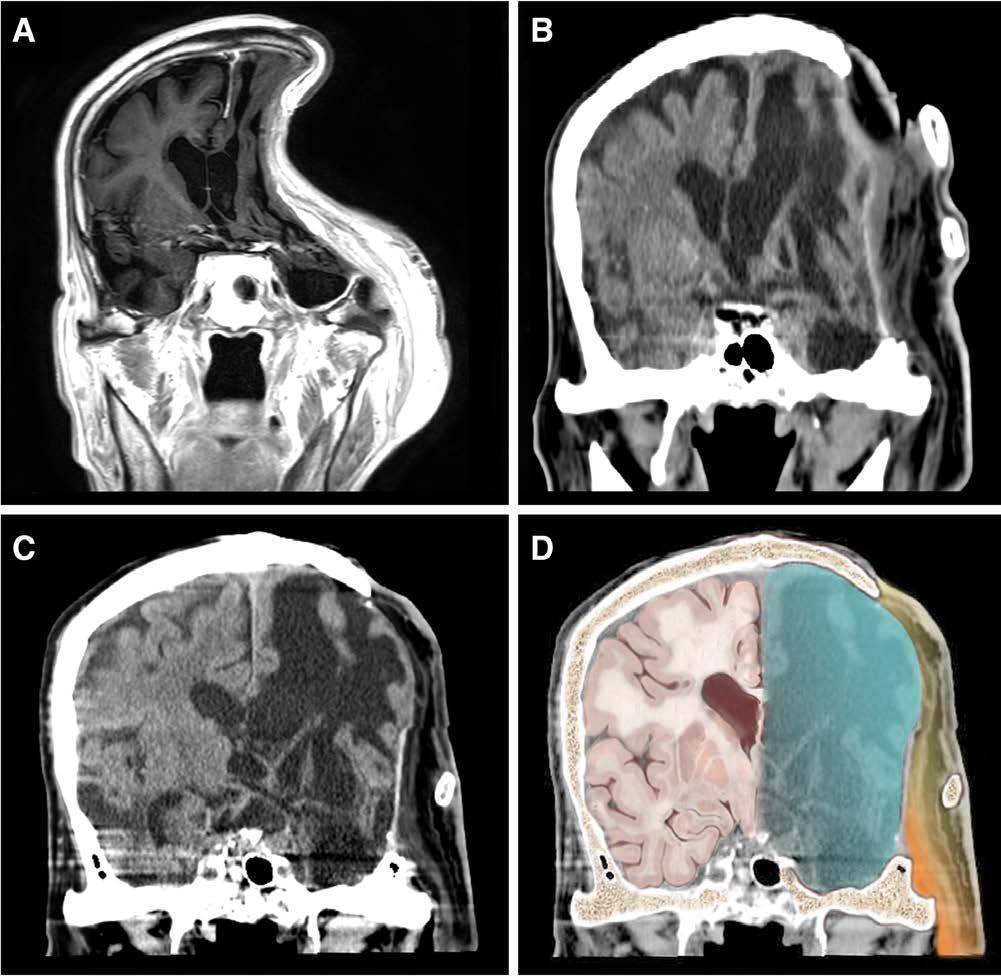
Comparison of coronal views obtained in different stages of the “kebab” technique. (A) Magnetic resonance image 2 months after the first stage of the “kebab” method, showing no fluid accumulation. (B) Nine weeks after the second stage of the “kebab” method. The intracranial volume is shown expanding against the atmospheric pressure after insertion of the rib grafts. (C) Unlabeled image at 2-year follow-up. (D) Labeled image demonstrating the latissimus dorsi myocutaneous flap (*yellow and orange*) and total reversal of sunken skin flap (*blue*).

We planned a two-stage reconstruction as follows: soft-tissue coverage to control recurrent infection and secondary bone grafting to reverse the depressed scalp (Fig. 2E). After implant removal and debridement (Fig. 1G and H), the calvarial defect was 15 × 15 cm in dimension, with a crescent-shaped scalp defect (Fig. 2A). The patient underwent left free LD myocutaneous flap reconstruction with an L-shaped skin paddle, covering the entire epidural dead space and most of the scalp tissue loss (Fig. 2B). The recipient vessels were the left facial vessels. The residual skin defect was covered with skin grafts. A 3-month follow-up brain magnetic resonance imaging revealed neither epidural fluid accumulation nor any evidence of infection, but the depressed contour was still evident (Fig. 3A).

Subsequently, we harvested his left eighth and ninth ribs, created subcutaneous dissection planes by W-shaped incisions, and fixated the rib grafts with screws in a crisscross fashion (Fig. 2C and D). Rib grafts were anchored to the LD muscle fascia with absorbable sutures. Postoperatively, the patient reported marked amelioration of headache and no flap compromise or donor-site morbidity (Fig. 2D). Five months after the reconstruction, the longitudinal rib graft was removed because of culture-negative bone exposure, and the wound was closed primarily.

### 2.3. Results

A 2-year postoperative brain CT scan revealed a totally reversed sunken skin flap, resolved midline shift, and a stable subarachnoid space (Fig. 3B–D). The patient was well without clinical signs of infection or headache until 3 years postoperatively.

## 3. Discussion

Postoperative infection is a significant cause of morbidity after cranioplasty.^[3]^ The decision to use alloplastic or autologous material is multifactorial. Several systematic reviews and meta-analysis have reported no significant difference in postoperative infection rates between allografts and autologous bone flaps.^[6,7]^ Allografts such as titanium or polyetheretherketones are widely used owing to their high durability, biocompatibility, and computer-aided design, but mesh exposure and skin necrosis are not uncommon.^[8]^ On the other hand, autologous tissue transfer with abundant volume and vasculature is indicated for complicated cranioplasty such as intolerance to foreign materials, recurrent infection, and soft-tissue insufficiency.^[9–12]^ However, one-stage cranioplasty with an osseous chimeric flap inevitably creates a dead space underneath the rigid bony structure, while intracranial dead space > 2 cm was associated with fluid accumulation and reconstructive failure.^[13]^ Early reconstruction before the subsidence of the infection often leads to devastating outcomes. Therefore, we proposed a novel two-stage reconstruction method called the “kebab” technique, which uses a free myocutaneous flap and delayed rib grafts, mimicking grilled meat threaded on to a skewer (Fig. 2E). This method can enhance bacterial clearance by the preemptive transfer of a well-vascularized myocutaneous flap, allow radiological monitoring during flap maturation, and prevent paradoxical brain herniation caused by delayed rib graft placement. Donor-site morbidity is minimized, and the operation is less strenuous than the chimeric flap reconstruction.

The “kebab” technique is innovative in successful combination of myocutaneous free flap and delayed bone grafts for recurrent infection and sinking skin flap syndrome. In the first stage of kebab cranioplasty, the role of the free myocutaneous flap is to enhance the penetration of antimicrobial agents and cover the exposed dura or intracranial content. Early expansion of the brain volume is usually not anticipated during this stage because of a lack of bony structures. If the infection cannot be overcome, bone grafts can be preserved for delayed reconstruction. The LD flap can provide large areas of thinner coverage with reliable anatomy.^[13,14]^ This workhorse flap has been combined with tissue expanders and alloplastic implants to salvage complicated cranioplasties.^[3]^ Factors such as pedicle length, risk of vascular kinking, and robustness of the vascular condition should be considered when selecting recipient vessels. The second stage of kebab cranioplasty aims to establish bony support for the soft tissue construct after resolution of the infection. Conceptually, a secondary procedure on a free flap is avoided owing to the risk of pedicle injury. Given that free myocutaneous flaps mature within one month, the safety of the delayed bone graft can be guaranteed by careful selection and inset.^[15]^ Rib grafts surpass calvarial bone grafts for our “kebab” technique because the latter was seldom used in larger defects (>200 cm^2^) or defects encompassing the frontotemporoparietal area.^[16]^ In addition, harvest and inset of a contralateral calvarial bone flap requires repositioning, longer incisions and wider dissection, which carry higher risks of dural injury and flap necrosis. In contrast, rib grafts can be harvested with a two-team approach in the same surgical position.^[17]^ Its natural curvature is preferable for calvarial defects than fibula or iliac bones. Elevation of non-vascularized ribs requires a shorter operative time and leaves most of the intercostal structures undisturbed.

The present case indicated that two perpendicularly aligned ribs were adequate to restore three-dimensional cranial capacity and provide an enduring dynamic effect (Fig. 3B–D). To maximize the osteoconduction and osteoinduction of non-vascularized bone grafts, we inserted full-thickness ribs into a well-vascularized recipient site and achieved rigid fixation with plates and screws.^[3,18]^ The onlay fixation can restore brain volume, but the relatively depressed region may be cosmetically suboptimal, especially for the non-hair-bearing area. Multiple rib grafts or split ribs with lattice work will provide better aesthetic outcome, but bone resorption and donor site morbidity should be taken into consideration. Postoperative pulmonary complications, such as pleural tear, atelectasis, pneumothorax, and pneumonia have been reported and require timely management and gradual respiratory training.^[19,20]^ Hematoma, wound infection and chronic pain may result in reoperation or prolonged hospitalization. In our approach to rib graft positioning, we opted for a crisscross arrangement because of its capacity to deliver solid three-dimensional support and facilitate ease of fixation. This design effectively counteracted the potential weakness inherent to the cantilever of curved grafts. The ideal number of rib grafts and the balance between cosmesis, donor-site morbidity and cerebral hemodynamics remain to be elucidated. Although the aesthetic outcome of “kebab” cranioplasty may be limited, the neurophysiological effect is comparable with that of custom-made implants (Fig. 3C and D). Cautious use of tissue expanders after adequate infection control may help to adjust the hairline and improve the cosmetic results. The present case is the first reported case of applying a combination of a free myocutaneous flap and delayed rib graft for refractory infection after multiple failed cranioplasties. Its unique design and advantages should be added to the armamentarium for calvarial reconstruction techniques.

## 4. Conclusions

Cranioplasty complicated with recurrent infection is always challenging. The “kebab” technique consists of 2 stages, free myocutaneous flap and delayed rib graft placements, and is suitable for patients with multiple cranioplasties and who are unable to withstand major reconstructions.

## Author contributions

**Conceptualization:** Liang-Jui Chiang, Jung-Shun Lee.

**Data curation:** Jing-Wei Lee, Po-Hsuan Lee.

**Investigation:** Liang-Jui Chiang, Po-Hsuan Lee.

**Writing – original draft:** Liang-Jui Chiang, Jing-Wei Lee.

**Writing – review & editing:** Jing-Wei Lee, Jung-Shun Lee.
